# Patterns of Nucleotide Diversity at the Regions Encompassing the *Drosophila Insulin-Like Peptide* (*dilp*) Genes: Demography vs. Positive Selection in *Drosophila melanogaster*

**DOI:** 10.1371/journal.pone.0053593

**Published:** 2013-01-07

**Authors:** Sara Guirao-Rico, Montserrat Aguadé

**Affiliations:** Departament de Genètica, i Institut de Recerca de la Biodiversitat, Universitat de Barcelona, Barcelona, Spain; The University of Queensland, St. Lucia, Australia

## Abstract

In Drosophila, the insulin-signaling pathway controls some life history traits, such as fertility and lifespan, and it is considered to be the main metabolic pathway involved in establishing adult body size. Several observations concerning variation in body size in the Drosophila genus are suggestive of its adaptive character. Genes encoding proteins in this pathway are, therefore, good candidates to have experienced adaptive changes and to reveal the footprint of positive selection. The Drosophila insulin-like peptides (DILPs) are the ligands that trigger the insulin-signaling cascade. In *Drosophila melanogaster*, there are several peptides that are structurally similar to the single mammalian insulin peptide. The footprint of recent adaptive changes on nucleotide variation can be unveiled through the analysis of polymorphism and divergence. With this aim, we have surveyed nucleotide sequence variation at the *dilp1-7* genes in a natural population of *D. melanogaster*. The comparison of polymorphism in *D. melanogaster* and divergence from *D. simulans* at different functional classes of the *dilp* genes provided no evidence of adaptive protein evolution after the split of the *D. melanogaster* and *D. simulans* lineages. However, our survey of polymorphism at the *dilp* gene regions of *D. melanogaster* has provided some evidence for the action of positive selection at or near these genes. The regions encompassing the *dilp1-4* genes and the *dilp6* gene stand out as likely affected by recent adaptive events.

## Introduction

In Drosophila, like in all holometabolous insect species, adult body size is mainly determined during the larval stages, as a product of both the growth rate and the duration of the growth period in each larval phase. Nutrition plays also a critical role in determining adult body size, since variation in caloric intake (quality and amount) in larval stages causes variation in growth rate, which in turn affects size at different larval stages. In insects, the insulin-signaling pathway is the main known metabolic pathway involved in establishing adult body size [1]–[4]. This pathway also plays a central role in fundamental biological processes such as metabolism, reproduction, aging and growth [4]–[6]. Multiple observations concerning variation in Drosophila adult body size are indicative of its adaptive character [7]–[23]. Moreover, life history traits such as fertility and lifespan generally reflect adaptive responses to environmental pressures and, thus, both positive and negative selection might have played an important role in the molecular evolution of the genes underlying such characters [24]. Genes involved in this pathway are, therefore, good candidates to have experienced adaptive changes and to reveal the footprint of positive selection. It has, indeed, been recently shown that the insulin-like receptor, which is the first component of the insulin-signaling pathway, has undergone adaptive change in its evolutionary past [25], [26].

The Drosophila insulin-like peptides (DILPs) are the ligands that trigger the insulin-signaling cascade. In *Drosophila melanogaster*, there are several *dilp* genes encoding proteins that are structurally similar to mammalian insulin. In *D. melanogaster*, five *dilp* genes are located on the 3L chromosomal arm at cytological position 67C8-9 (genes *dilp1-5*) whereas genes *dilp6* and *dilp7* are on the X chromosome at cytological positions 3A1 and 3E2, respectively. The autosomal genes consist of a cluster with four contiguous genes (*dilp1-4*) and a fifth gene (*dilp5*) separated from the rest by one intervening gene (figures S1 and S2). Over the last decade, the isolation and characterization of diverse *D. melanogaster* mutants for the *dilp* genes has provided experimental support for the involvement of these genes in regulating body size [27]–[29]. *dilp* genes are independently transcriptionally regulated in response to nutrition, as well as in a tissue- and stage-specific manner during development [27], [30]. It has been shown that genes *dilp1*, *dilp2* and *dilp3* are consecutively expressed during larval stages, whereas *dilp 6* controls growth specifically during the pupal stage. Among *dilp* genes, *dilp2* is the most potent growth promoter and *dilp3* is the gene most responsive to diet changes [29]. It has also been shown that DILPs can act redundantly [29]. There is, therefore, some evidence for both functional differentiation and functional redundancy among DILPs.

In new environments, like those encountered by *D. melanogaster* in its colonization and expansion through Europe, there would be ample opportunities for selection to have acted either separately or jointly on *dilp* genes. Moreover, the out-of-Africa expansion of the species imposed demographic changes during this process. In order to detect the putative action of positive selection in this very recent past of *dilp* genes, we surveyed nucleotide variation at each of the *dilp* genes in a European population of *D. melanogaster*, because levels and patterns of polymorphism can be informative on recent adaptive changes. We also compared levels of polymorphism and divergence from *D. simulans* at synonymous and nonsynonymous sites of coding regions, because this comparison can be informative of adaptive amino acid replacements after the species split. These analytical approaches capture different aspects of the footprint left by positive selection on DNA sequences, and in the window of evolutionary time in which they are able to detect this footprint.

Our comparison of polymorphism and divergence at synonymous and nonsynonymous sites at the *dilp* genes has provided no evidence for amino acid adaptive substitutions in any of the DILPs since the split of *D. melanogaster* and *D. simulans*. In contrast, our survey of polymorphism at *dilp* gene regions of *D. melanogaster* has provided some evidence for the recent action of positive selection at or near these genes. Indeed, the *dilp1-4* region exhibited a significant excess of high-frequency derived variants (as indicated by a highly negative *H* value), whereas the spatial pattern of variation at the *dilp6* region had a significantly better fit to a selective than to a non-selective model.

## Materials and Methods

### Drosophila Strains

Twelve isochromosomal lines for each the third chromosome (CNIII) and the X chromosome (CNX) that had been extracted from a natural population of *Drosophila melanogaster* (Sant Sadurní d’Anoia, Barcelona, Spain) were used to obtain the sequences of the *dilp* genes located on the third (*dilp1-5*) and X (*dilp6* and *dilp7*) chromosomes, respectively. These lines were obtained and kindly provided by D. Orengo [31]. In addition, two highly inbred *Drosophila simulans* lines –SAL and VSAL from a natural population in Alella (Barcelona, Spain) obtained by 10 generations of sib mating– were used to sequence the autosomal *dilp* genes, whereas one X-isochromosomal line –MO [32] from a natural population in Montblanc (Tarragona, Spain)– was used to sequence the X-linked genes.

### Polymerase Chain Reaction (PCR) Amplification and Sequencing

DNA was extracted from 1 and 10 individuals (in case of highly inbred lines and isochromosomal lines, respectively) using either a modification of protocol 48 in Ashburner [33] or the Puregene DNA purification kit (Puregene, Gentra systems). The Oligo version 4.1 program [34] was used to design oligonucleotides, for both PCR amplification and DNA sequencing, based on the *dilp* genes sequences retrieved from Flybase [35] available at http://flybase.bio.indiana.edu/. The amplification products were purified either with a single-strand DNA enzymatic hydrolysis reaction (“ExoSAP-IT” method, USB), or with Amicon Microcon-PCR columns (Millipore). All fragments were cycle sequenced using the ABI PRISM Dye Terminator Cycle Sequencing Ready Reaction kit (Applied Biosystems) and subsequently separated on ABI PRISM 3700 automated DNA sequencer (ABI Applied Biosystems). DNA sequences were obtained on both strands for each line. All new sequences in this article have been deposited in the EMBL database under the accession numbers HE654131-HE654180.

### DNA Sequence Analysis

For each line and sequenced region, the DNA sequences were assembled using the SeqMan version 5.53 program (DNASTAR, Madison, WI). Sequences were multiply aligned and manually edited using the ClustalW program [36] and the MacClade version 3.05 program [37], respectively. Intraspecific and interspecific analyses were performed using the DnaSP version 5.10.01 program [38]. Nucleotide polymorphism was estimated by the number of segregating sites (*S*), nucleotide diversity (*π*; [39]), the number of haplotypes (*h*) and haplotype diversity (*Hd*; [39]). Interspecific divergence was estimated as the number of nucleotide substitutions per site (*K*). Synonymous (*Ks*) and nonsynonymous (*Ka*) divergence was estimated with the Nei and Gojobori method [40], and subsequently corrected according to Jukes and Cantor [41].

### Neutrality Tests

The multilocus HKA test ([42]; J. Hey, http://lifesci.rutgers.edu/~heylab/index.html) was used to evaluate the putative heterogeneity across regions of the polymorphism to divergence ratio. Confidence intervals were established via coalescent simulations as well as using the Chi-square approximation. The McDonald and Kreitman (MK) test [43] was performed to examine whether the ratio of polymorphic to fixed changes was similar at synonymous and nonsynonymous sites.

The Tajima [44] and Fay and Wu [45], [46] tests were conducted to examine whether the frequency spectrum of polymorphic nucleotide mutations conformed to neutral expectations. For each gene or sequenced region, the orthologous *D. simulans* sequence was used as outgroup in the normalized Fay and Wu *H* test, which is based on the unfolded frequency spectrum. Monte Carlo simulations based on the coalescent process [47] were carried out to obtain the *P*-values of these tests both under the standard neutral model (hereafter SNM), and under a previously described bottleneck model [48], [49]. Confidence intervals were established from the distribution of each test statistic obtained from 1000 simulated replicates. All simulations were carried out using the mlcoalsim version 1 program [50]. Simulations were performed fixing the number of segregating sites (*S*) and taking into account the uncertainty of *θ* ([51]; Rejection Algorithm method, RA), and with the possibility of multiple hits. The simulations were performed using a uniform prior distribution of *θ* values (ranging from 0.001 to 0.06) and two estimates of the population recombination rate –RM, which is obtained from the comparison of the physical and genetic maps ([52]; Release 5.19 of the *Drosophila melanogaster* Recombination Rate Calculator)–, and R0.25 =  RM/4 as an intermediate value of R.

### Composite Likelihood Ratio (CLR) Test and Goodness-of Fit (GOF) Test

The statistical composite likelihood method implemented in the clsw program [53] was used to evaluate whether there is any evidence for a recent selective event (hitchhiking effect) in any of the four regions studied. We applied test B, which uses the level of variation estimated from the data (*θ_W_*; [54]) to calculate the likelihood, and option 1, which uses information from an outgroup (*D. simulans*) to distinguish between ancestral and derived alleles. Significance was established through comparison of the CLR value obtained from the data to the CLR values obtained from simulations under the SNM and also under a bottleneck model [48], [49]. When the CLR test revealed a better fit of the data to the selective sweep than to both the SNM model and the bottleneck (BN) model, the GOF test [55] was performed to discriminate false positives. In this test, the null model is the selective sweep model and the alternative model is a general model in which the number of sequences at each position that carry the derived mutation are binomially distributed. The null distribution was obtained by applying the GOF test to a simulated data set obtained under the selective sweep model using the mlcoalsim version 1 program [50].

## Results

### Polymorphism in *Drosophila Melanogaster*

Nucleotide polymorphism was estimated both for the four *dilp* regions (table 1, tables S1 and S2) and for each of the seven *dilp* genes (table 2). A total of 204 segregating sites (figures S1, S2, S3) were detected with singletons in over half of the sites (i. e., 113 out of 204). The estimated level of nucleotide diversity varied from 0.003 to 0.008 for *π*_total_ and from 0.003 to 0.011 for *π*_silent_. Table 2 summarises nucleotide polymorphism at different functional classes of the *dilp* genes. A total of 65 segregating sites were detected in these genes, 42 of which were singletons. Levels of silent nucleotide diversity were similar at the *dilp2*, *3*, *4*, and *7* genes (table 2). These estimates were approximately one order of magnitude higher than those at the *dilp1* and *dilp5* genes. Synonymous sites were in general more polymorphic than intronic sites. Estimates of synonymous nucleotide variation at the *dilp2*, *3*, *4* and *7* genes were similar to the average values reported for other genes in this species (*π_s_*  = 0.0134, [56]; *π_s_*  = 0.0165, [57]), and one order of magnitude higher than estimates at the *dilp1*, *dilp5* and *dilp6* genes. Additionally, levels of silent polymorphism were more variable at both flanking and intergenic regions than at coding regions (varying from 0.0002 to 0.012 vs 0.001 to 0.012, respectively; tables S1 and S2). Nonsynonymous nucleotide diversity (*π_a_*) was low in all genes except *dilp1*, which exhibited similar levels of synonymous and nonsynonymous nucleotide diversity. Indeed, DILP1 was the most variable DILP protein, with 5 amino acid polymorphisms (figure S4), 4 located in the C peptide and 1 in the B chain. The DILP6 and DILP7 proteins were the only other polymorphic proteins, each with a single polymorphic residue in the A chain.

**Table 1:** Nucleotide polymorphism and divergence at the four *dilp* gene regions.

	*dilp1-4* 1	*dilp5*	*dilp6* 2	*dilp7*
**No. sites**				
Silent^3^	6186.7	2960.2	2106.8	334.1
Total	7424	3201	2352	699
***S***				
Silent	91 (77)	66 (12)	27 (13)	13 (5)
Total	96 (81)	66 (12)	28 (14)	14 (6)
***π***				
Silent	0.003	0.009	0.004	0.011
Total	0.003	0.008	0.003	0.005
***h***	10	10	12	9
***Hd***	1.000	0.970	1.000	0.910
**K**				
Silent	0.059	0.051	0.061	0.164
Total	0.052	0.048	0.056	0.073

1 Sample size was 12 for all regions except *dilp1-4* (10).

2 The sequenced region consist of two fragments separated by a ∼390-bp stretch located at the first intron of the *dilp6* gene.

3 Silent refers to variation at non-coding sites and at synonymous sites of coding regions.

*S*, number of segregating sites (number of singletons in parentheses); *π*, nucleotide diversity; *h*, number of haplotypes; *Hd*, haplotype diversity; *K*, nucleotide divergence.

**Table 2:** Nucleotide polymorphism and divergence at the seven *dilp* genes.

	*dilp1*	*dilp2*	*dilp3*	*dilp4*	*dilp5*	*dilp6*	*dilp7*
**No. sites**							
Intronic	0	73	72	61	71	870	170
Synonymous	110.3	98.5	85.8	103.1	80.2	75.8	112.1
Silent^1^	110.3	171.5	157.8	164.1	151.2	1312.8	282.1
Nonsynonymous	351.7	312.5	274.2	298.9	240.8	245.2	364.9
Total	462	484	432	463	392	1558	647
***S***							
Intronic	n. a.	2 (1)	2 (1)	0	0	18 (10)	5 (0)
Synonymous	1 (1)	6 (6)	6 (5)	7 (6)	1 (1)	0	6 (4)
Silent	1 (1)	8 (7)	8 (6)	7 (6)	1 (1)	22 (11)	11 (4)
Nonsynonymous	5 (4)	0	0	0	0	1 (1)	1 (1)
Total	6 (5)	8 (7)	8 (6)	7 (6)	1 (1)	23 (12)	12 (5)
***π***							
Intronic	n. a.	0.008	0.008	0	0	0.006	0.010
Synonymous	0.002	0.012	0.016	0.015	0.002	0	0.012
Silent	0.002	0.010	0.012	0.009	0.001	0.005	0.011
Nonsynonymous	0.003	0	0	0	0	0.0007	0.0005
Total	0.003	0.004	0.004	0.003	0.0004	0.004	0.005
***K***							
Intronic	n. a.	0.082	0.054	0.051	0.030	0.062	0.166
Synonymous	0.116	0.143	0.197	0.159	0.094	0.177	0.118
Silent	0.116	0.116	0.133	0.117	0.064	0.071	0.143
Nonsynonymous	0.031	0.010	0.007	0.020	0.008	0.021	0.010
Total	0.051	0.046	0.049	0.053	0.029	0.062	0.059
***ω***	0.270	0.070	0.037	0.128	0.089	0.120	0.084

1 Silent refers to variation at non-coding sites and at synonymous sites of coding regions.

*S*, number of segregating sites (number of singletons in parentheses); *π*, nucleotide diversity; *K*, nucleotide divergence; *ω*, *K_a_*/*K_s_* ratio; n. a., not applicable.

### Divergence between *Drosophila Melanogaster* and *Drosophila Simulans*

The sequences newly obtained for the four regions spanning the seven *dilp* genes in *D. simulans* were used to estimate nucleotide divergence between this species and *D. melanogaster* both for the four *dilp* regions (table 1) and for each *dilp* gene (table 2). Levels of nucleotide divergence at the *dilp* regions, when considering all sites and only silent sites, were similar for all regions except for the *dilp7* region, which exhibited the highest level of divergence (table 1). Among genes, *dilp5* showed the lowest silent divergence estimate (table 2). The estimates of synonymous divergence (*K_s_*) were similar to the average values reported previously for other genes between these species (*K_s_*  = 0.11, [58]; *K_s_*  = 0.11, [59]; *K_s_*  = 0.13, [60]) except at genes *dilp5* and *dilp6*. At the *dilp* genes, nucleotide divergence was more heterogeneous at nonsynonymous sites (*K_a_*) than at synonymous sites (table 2). The ratio of nonsynonymous to synonymous divergence (*ω* = *K_a_*/*K_s_*) was <1 in all cases (table 2), reflecting the action of purifying selection against nonsynonymous changes. Similarly to *K_a_* estimates, the *ω* estimates varied among genes (table 2). Amino acid divergence between the *D. melanogaster* and *D. simulans* DILPs (with 28 amino acid replacements; figure S4) also varied among peptides. Indeed, divergence at DILP1, DILP4 and DILP6 (with 8, 6 and 5 amino acid changes, respectively) was higher than at the rest of DILPs. Moreover, most of the detected amino acid replacements (25 out of 28) were located either in the signal peptide or in the C peptide, which might be indicative of weaker purifying selection acting on these protein domains.

### Polymorphism and Divergence: the HKA and MK Tests

The multilocus HKA test ([42]; J. Hey, http://lifesci.rutgers.edu/~heylab/index.html) was used to evaluate whether the levels of silent nucleotide polymorphism and divergence at the *dilp* genes were correlated. No significant heterogeneity in the polymorphism to divergence ratio was detected. We also conducted the MK test [43] that compares the amount of variation within and between species at two different types of sites (synonymous and nonsynonymous). No significant departure from the proportionality between polymorphism and divergence expected under the SNM was detected at any of the seven *dilp* genes, either when changes fixed between *D. melanogaster* and *D. simulans* or when the *D. melanogaster* lineage-specific fixed changes, were considered. There is no evidence, therefore, for adaptive protein evolution of the DILP proteins since the *D. melanogaster* and *D. simulans* split.

### Patterns of Nucleotide Diversity: Tajima’s *D* and Fay and Wu’s *H* Test Statistics

Tajima *D*’s [44] and Fay and Wu *H*’s [45] test statistics were calculated separately for each of the four regions studied (table 3). *D* values were negative for 3 regions (*dilp1-4*, *dilp6* and *dilp7*), whereas *H* values were negative for 2 (*dilp1-4* and *dilp5*). For 3 regions (*dilp5*, *dilp6* and *dilp7*), Tajima’s *D* and Fay and Wu’s normalized *H* values did not depart significantly from expectations of either the SNM or the bottleneck (BN) model, irrespective of the recombination rate estimate used. For the fourth region (*dilp1-4*), the Tajima test and the Fay and Wu test revealed significant departures from SNM expectations, which would indicate a significant excess of high-frequency derived variants (table 3). However, under the most realistic bottleneck scenario, only the estimated *H* value remained significant (table 3). According to the test results, this excess cannot be solely due to the demographic history of the population studied. Indeed, when the observed *D* and *H* values were compared to the corresponding empirical distributions obtained from multilocus surveys of variation in the same population, for either X-linked or autosomal loci ([61], unpublished data), only the *H* value estimated for the *dilp1-4* region fell within the bottom 5% of the empirical Fay and Wu’s *H* distribution.

**Table 3:** Neutrality test of the *dilp* gene regions.

	Tajima’s *D*				Fay and Wu’s *H*			
Region	*D* value	R	SNM	BN	*H* value	R	SNM	BN
***dilp1-4***	−1.72	R_M_	**<0.001**	0.11	−3.55	R_M_	**<0.001**	**0.01**
		R_0.25_	**<0.001**	0.22		R_0.25_	**<0.001**	**0.04**
***dilp5***	0.87	R_M_	0.99	0.68	−0.23	R_M_	0.36	0.94
		R_0.25_	0.98	0.60		R_0.25_	0.81	0.85
***dilp6***	−0.59	R_M_	0.08	0.91	0.06	R_M_	0.57	0.84
		R_0.25_	0.16	0.93		R_0.25_	0.73	0.85
***dilp7***	−0.71	R_M_	0.13	0.48	0.44	R_M_	0.78	0.90
		R_0.25_	0.21	0.49		R_0.25_	0.66	0.94

R, population recombination rate per nucleotide (see Materials and Methods); *D*, Tajima’s *D*; *H*, normalized Fay and Wu’s *H*; SNM, standard neutral model; BN, bottleneck model. Statistically significant values are indicated in bold.

### The Composite Likelihood Ratio (CLR) Test and the Goodness-of-fit (GOF) Test: Sweep Detection and Localization by Maximum Likelihood

A composite-likelihood method was used to distinguish selective sweeps from stochastic neutral variation [53]. The CLR test was applied separately to each of the four regions studied: the *dilp1-4* gene cluster and the *dilp5*, *dilp6* and *dilp7* regions (table 4). This analysis yielded a significant better fit of variation at the *dilp1-4* gene cluster to the selective sweep model than to the SNM. Variation at the *dilp5* and *dilp6* regions also exhibited a better fit to the selective sweep model than to the SNM but unlike at the *dilp1-4* region, only under a particular recombination rate value (RM and R0.25 values, respectively). For each *dilp* region, the observed CLR values were additionally tested against the null distribution built from bottleneck simulations in order i) to evaluate the possibility of false positives in cases where the CLR test yielded significant results, and ii) to confirm that for those regions that conformed to the SNM predictions, the results were robust to demographic change. This analysis yielded a significant better fit of variation at the *dilp1-4* gene cluster to the selective sweep model than to the bottleneck model, irrespective of the recombination rate estimate used. Also, variation at the *dilp6* region showed a better fit to the selective sweep model than to the bottleneck model but only under the R0.25 recombination rate value (table 4). Estimates of the strength of selection and of the location of the putative target of selection (using the clsw program) for each the *dilp1-4* and *dilp6* regions were used to perform the GOF test, which allows further discrimination of false positives. Only for the *dilp6* region under the R0.25 recombination value, this test fails to show a significantly better fit to the more general model than to the selective model (*P*-value  = 0.214).

**Table 4:** Composite likelihood ratio test and Goodness-of-fit test.

Region	*L* 1	*S* 1	R		SNM	BN	*a*	*X*	GOF
***dilp1-4***	7534	93	R_M_	0.063	**<0.001**	**0.007**	3461.55	10	0.035
			R_0.25_	0.016	**<0.001**	**0.005**	716.30	26	0.041
***dilp5***	3266	56	R_M_	0.063	**0.010**	0.288	n. a.	n. a.	n. a.
			R_0.25_	0.016	0.051	0.197	n. a.	n. a.	n. a.
***dilp6^a^***	2378	24	R_M_	0.047	0.293	0.071	n. a.	n. a.	n. a.
			R_0.25_	0.012	**0.008**	**0.001**	22.76	2604	**0.214**
***dilp7***	710	11	R_M_	0.058	0.892	0.968	n. a.	n. a.	n. a.
			R_0.25_	0.014	0.758	0.952	n. a.	n. a.	n. a.

1 Sites with missing or ambiguous information in the outgroup were excluded from this analysis.

*L*, number of nucleotides of the multiply alignment region; *S*, number of segregating sites; R, population recombination rate per nucleotide (in 2N units); SNM, standard neutral model; BN, bottleneck model; α strength of positive selection (in 2Ns units); *X*, location of the target of positive selection; GOF, goodness-of-fit test; n. a., not applicable. For the GOF test, probability values that do not support a better fit to the general alternative model than to the selective sweep model are indicated in bold.

## Discussion

Changes in the biotic and abiotic environment of organisms promote adaptation, i.e., evolutionary change driven by positive selection. Even if populations may be constantly exposed to environmental change, it is easy to visualize certain scenarios, like the range expansion of a species, in which a population encounters a higher than average degree of change. These scenarios can be considered candidates for the species and/or populations involved to have experienced bursts of adaptive change. It is for this reason that surveys of nucleotide variation often target derived populations.

In Drosophila, the multilocus analysis of polymorphism and divergence at coding regions has revealed that ∼50% of the amino acid substitutions detected between closely related species had been driven by positive selection [62], [63]. Moreover, the comparison of coding and non-coding regions similarly has revealed that adaptive changes at non-coding regions might have been considerably common in the evolution of *Drosophila melanogaster* [64]. Application of the MK test [43] to the 7 *dilp* coding regions has provided no evidence for adaptive amino acid substitutions in any of the DILP peptides since the split of the *D. melanogaster* and *D. simulans* lineages. A similar study on the insulin receptor, a transmembrane receptor with an extracellular part that binds insulin and a cytosolic part with signal-transduction capacities, was previously conducted [25]. Indeed, this study yielded a negative result for the extracellular part of the receptor, but not for its cytosolic part, which together with the present result would indicate that selection had not favoured changes in the ligand-receptor (DILP-InR) interaction but on the signal-transduction capacity of the receptor upon its activation by the ligand.

*Drosophila melanogaster* is a cosmopolitan species that originated in central Africa and later expanded its distribution area worldwide [65]. European populations of this species are non-stationary derived populations, as confirmed by multilocus analysis of variation at non-coding regions [31], [66], [67]. These surveys revealed that a simple bottleneck scenario could explain, despite not completely, the pattern of variation detected at these regions [31], [48], [49], [66], [67], but see also [68], [69]. Moreover, they provided estimates for the parameters of the proposed bottleneck model, which can thereafter be used in hypothesis testing. Indeed, this approach has already led to the identification of a few regions that were the targets of recent selective events [70]–[74].

The present survey of polymorphism at the regions encompassing the *dilp* genes in *D. melanogaster* has provided some evidence for the recent action of positive selection at or near some of these genes, more specifically at the *dilp1-4* and *dilp6* regions. Indeed, the pattern of variation at the *dilp1-4* region exhibited a significant excess of high-frequency derived variants (as indicated by the highly negative *H* value) relative not only to expectations of the SNM and the more realistic bottleneck model (table 3) but also when compared to the corresponding empirical distributions of the *H* test statistic obtained from multilocus surveys of variation in the same population ([31], unpublished data). Although the Kim and Stephan test [53] also yielded a significant result for this region when using bottleneck simulations, this result was not clearly supported by the GOF test. In contrast to these results for the *dilp1-4* region, the frequency spectrum at the *dilp6* region did not depart from bottleneck expectations, whereas the GOF test for this region clearly supported the significant result of the Kim and Stephan test under an intermediate level of recombination (but not under the higher level estimated from the genetic map). The dependency of the Kim and Stephan test result on the level of recombination clearly points to the need for accurate estimates of this parameter and therefore for new experimental efforts using a large and dense set of markers to obtain fine-scale genetic maps [75], [76]. There is also some degree of uncertainty in the effect of the particular bottleneck scenario considered on our conclusions [77]. In summary, present estimates of the level and pattern of polymorphism at the *dilp* genes do not provide strong evidence for recent adaptive changes either in the genes themselves or in their vicinity, although the *dilp1-4* and *dilp6* regions stand out as likely affected by such events.

It is worth noting that our population-genetic analysis has unveiled the footprint of positive selection at the *dilp1-4* cluster region and the *dilp6* region. These regions encompass four of the genes (*dilp1*, *dilp2, dilp3* and *dilp6*) that are involved in establishing adult body size by promoting growth at the larval and pupal stages, with one of them (*dilp3*) also involved in the response to nutritional changes [29]. The signals detected at the *dilp6* gene and at the *dilp1-4* cluster might reflect that gene *dilp6* and at least one of the genes in the cluster might have been the target of selection acting on their distinct functional roles. Selection might have also acted on DILP copies with partially redundant functions as a way to downplay stochastic variations in DILP synthesis or secretion in response to varying external conditions [78]. The out-of-Africa expansion of *D. melanogaster* exposed the colonizing populations to new environmental physical conditions as well as to new food sources. Selective pressures resulting from the flies’ exposure to these new environments led to many adaptive changes, among which those in adult body size and response to nutritional conditions might have targeted genes in the insulin-signaling pathway.

## Supporting Information

Figure S1 **(A)** Genomic organization of the *dilp1-4* gene region of *D. melanogaster*. Genomic DNA is represented by a line. The black arrow head points to the centromere. In genes, arrows indicate the direction of transcription. Colored boxes indicate exons of *dilp* genes. Introns are represented by a V symbol. **(B)** Nucleotide polymorphism at the *dilp1-4* gene region of *D. melanogaster*. The last row shows nucleotide information present in *D. simulans* for each polymorphic site detected in *D. melanogaster*. *, nonsynonymous polymorphism. Dots indicate nucleotide variants identical to the first sequence and dashes indicate gaps. d, deletion; i, insertion; E, exon.
(PDF)

Figure S2 **(A)** Genomic organization of the *dilp5* gene region of *D. melanogaster*. Genomic DNA is represented by a line. The black arrow head points to the centromere. In gene, arrow indicates the direction of transcription. Colored boxes indicate exons of *dilp5* gene. Intron are represented by a V symbol. **(B)** Nucleotide polymorphism at the *dilp 5* gene region of *D. melanogaster*. The last row shows nucleotide information present in *D. simulans* for each polymorphic site detected in *D. melanogaster*. Dots indicate nucleotide variants identical to the first sequence and dashes indicate gaps. d, deletion; i, insertion; E, exon.
(PDF)

Figure S3 **(A)** Genomic organization of the *dilp6* and *dilp7* gene regions of *D. melanogaster*. Genomic DNA is represented by a line. The black arrow head points to the centromere. In genes, arrows indicate the direction of transcription. Colored boxes indicate exons of *dilp* genes Introns are represented by a V symbol. **(B)** Nucleotide polymorphism at the *dilp6* and *dilp7* gene regions of *D. melanogaster*. The last row shows nucleotide information present in *D. simulans* for each polymorphic site detected in *D. melanogaster*. *, nonsynonymous polymorphism. Dots indicate nucleotide variants identical to the first sequence and dashes indicate gaps. i, insertion; E, exon.
(PDF)

Figure S4 **(A)** Schematic representation of the predicted structure of the DILP1-5 proteins of *D. melanogaster*. SP, signal peptide; B, B chain; C, C peptide; A, A chain. The active peptide chains are denoted by colors. **(B)** Amino acid polymorphism in *D. melanogaster* and amino acid replacements between *D. melanogaster* and *D. simulans* at the DILP1-5 proteins. The last row shows the amino acid present in *D. simulans* for each polymorphic site detected in *D. melanogaster* and also for the sites with fixed differences between species. Dots indicate amino acid variants identical to the first sequence and dashes indicate deletions.
(PDF)

Figure S5 **(A)** Schematic representation of the predicted structure of the DILP6 and DILP7 proteins of *D. melanogaster*. SP, signal peptide; B, B chain; C, C peptide; A, A chain. The active peptide chains are denoted by colors. **(B)** Amino acid polymorphism in *D. melanogaster* and amino acid replacements between *D. melanogaster* and *D. simulans* at the DILP6 and DILP7 proteins. The last row shows the amino acid present in *D. simulans* for each polymorphic site detected in *D. melanogaster* and also for the sites with fixed differences between species. Dots indicate amino acid variants identical to the first sequence and dashes indicate deletions.
(PDF)

Table S1 **Nucleotide polymorphism and divergence at the autosomal dilp1-4 and dilp5 gene regions.**
(PDF)

Table S2 **Nucleotide polymorphism and divergence at the X-linked dilp6 and dilp7 gene regions.**
(PDF)
