# Supplementary material for: Patterns of Nucleotide Diversity at the Regions Encompassing the Drosophila Insulin-Like Peptide (dilp) Genes: Demography vs. Positive Selection in Drosophila melanogaster
Source: PLoS One. 2013 Jan 7;8(1):e53593. doi: 10.1371/journal.pone.0053593 (PMC3538593; doi:10.1371/journal.pone.0053593)
Supplement: Figure S1 — (A) Genomic organization of the dilp1-4 gene region of D. melanogaster. Genomic DNA is represented by a line. The black arrow head points to the centromere. In genes, arrows indicate the direction of transcription. Colored boxes indicate exons of dilp genes. Introns are represented by a V symbol. (B) Nucleotide polymorphism at the dilp1-4 gene region of D. melanogaster. The last row shows nucleotide information present in D. simulans for each polymorphic site detected in D. melanogaster. *, nonsynonymous polymorphism. Dots indicate nucleotide variants identical to the first sequence and dashes indicate gaps. d, deletion; i, insertion; E, exon. (PDF) [file pone.0053593.s001.pdf]

A

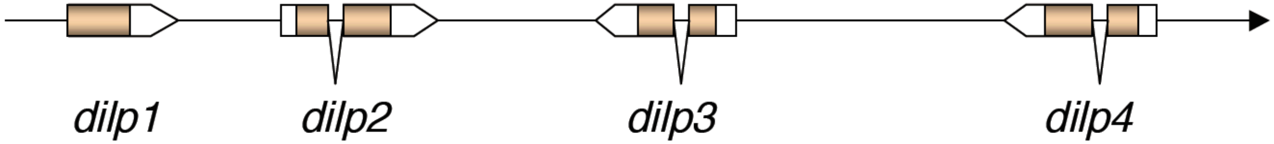

B

	91:i2	321	349:d14	363:i5	368:d2	370	371:d7	1047 * (E1)	1087 (E1)	1203 * (E1)	1211 * (E1)	1226 * (E1)	1236 * (E1)	1753	1755	1761	1784	1844	1979	2051	2136	2209	2261	2410 (E1)
**CNIII 1**	-	C	d	-	d	-	d	T	G	A	A	G	G	T	A	T	T	C	A	C	C	T	C	T
**CNIII 5**	-	A	T	A	C	C	T	.	A	.	.	C	.	C	G	C	G	T	.	.	.	G	T	.
**CNIII 6**	G	.	d	-	d	-	d	.	A	.	.	C	.	C	G	C	G	T	.	T	.	G	T	.
**CNIII 7**	-	.	d	-	d	-	d	.	A	.	.	C	.	C	G	C	G	T	.	.	.	G	T	.
**CNIII 15**	-	.	d	-	d	-	d	.	A	.	.	C	.	C	G	C	G	T	.	.	.	G	T	.
**CNIII 16**	-	.	d	-	d	-	d	.	A	.	.	C	.	C	G	C	G	T	.	.	.	.	.	.
**CNIII 18**	-	.	d	-	d	-	d	C	A	G	G	.	C	C	G	C	G	T	G	.	.	G	.	.
**CNIII 22**	-	.	d	-	d	-	d	.	A	.	.	C	.	C	G	C	G	T	.	.	T	.	.	.
**CNIII 35**	-	.	d	-	d	-	d	.	A	.	.	C	.	C	G	C	G	T	.	.	.	G	T	.
**CNIII 36**	-	.	d	-	d	-	d	.	A	.	.	C	.	C	G	C	G	T	.	.	.	G	T	C
D. simulans	**-**	**.**	**T**	**-**	**C**	**-**	**T**	**C**	**.**	**.**	**G**	**.**	**.**	**C**	**G**	**C**	**G**	**.**	**G**	**.**	**.**	**G**	**.**	**.**

	2488	2520	2555 (E2)	2603 (E2)	2609 (E2)	2612 (E2)	2660 (E2)	2788	2822	2917	2947	2967	2985	3019	3074	3168	3170:d2	3189	3195:d20	3215:d10	3236:d2	3262	3271	3277	3327
**CNIII 1**	C	T	T	C	A	G	A	C	C	T	C	G	C	T	T	A	d	C	T	d	d	A	T	T	G
**CNIII 5**	A	.	.	.	.	.	.	.	.	.	.	C	T	A	A	.	d	A	.	d	d	.	.	.	.
**CNIII 6**	A	.	.	.	.	.	.	.	.	.	G	C	T	A	A	.	d	A	.	d	d	.	.	.	.
**CNIII 7**	A	.	.	.	.	.	.	.	.	.	.	C	T	A	A	.	d	A	.	d	d	.	.	.	.
**CNIII 15**	A	C	C	.	.	.	C	T	G	C	.	C	T	A	A	.	d	A	.	d	d	.	.	.	.
**CNIII 16**	A	.	.	.	.	.	.	.	.	C	.	C	T	A	.	.	A	A	d	T	A	C	A	C	C
**CNIII 18**	A	.	.	.	.	.	.	.	.	.	.	C	T	A	A	.	d	A	.	d	d	.	.	.	.
**CNIII 22**	.	.	.	T	C	A	.	.	.	C	.	C	T	A	A	.	d	A	.	d	d	.	.	.	.
**CNIII 35**	A	.	.	.	.	.	.	.	.	.	.	C	T	A	A	T	d	A	.	d	d	.	.	.	.
**CNIII 36**	A	.	.	.	.	.	.	.	.	.	.	C	T	A	A	.	d	A	.	d	d	.	.	.	.
D. simulans	**.**	**C**	**C**	**T**	**C**	**.**	**C**	**T**	**.**	**.**	**.**	**C**	**.**	**A**	**.**	**.**	**A**	**A**	**.**	**T**	**A**	**-**	**.**	**.**	**C**

	3331	3366	3384	3418	3422	3428	3432	3437	3542	3608	3630	3631	3634:i	3635:d4	3637	3638	3639:i3	3642:d6	3648:i3	3666	3685	3709:i	3710	3716
**CNIII 1**	T	C	C	A	G	A	A	A	C	A	A	C	T	C	C	G	-	C	C	A	T	A	A	A
**CNIII 5**	.	.	.	.	.	.	.	.	.	.	.	.	.	.	.	.	-	.	.	.	.	.	.	.
**CNIII 6**	.	.	.	.	.	.	.	.	.	.	.	.	.	.	.	.	-	.	.	.	.	.	.	.
**CNIII 7**	.	.	.	.	.	.	.	.	.	.	.	.	.	.	.	.	-	.	.	.	.	.	.	.
**CNIII 15**	.	.	.	.	.	.	.	.	.	.	.	.	.	.	.	.	-	.	.	.	.	.	.	.
**CNIII 16**	A	T	A	T	A	G	T	C	.	G	T	A	.	G	T	A	A	.	.	.	.	.	.	.
**CNIII 18**	.	.	.	.	.	.	.	.	.	.	.	.	.	.	.	.	-	.	.	.	.	.	.	.
**CNIII 22**	.	.	.	.	.	.	.	.	d	.	.	.	.	.	.	.	-	.	.	.	.	.	.	.
**CNIII 35**	.	.	.	.	.	.	.	.	.	.	.	.	-	d	—	—	-	d	-	d	C	T	T	G
**CNIII 36**	.	.	.	.	.	.	.	.	.	.	.	.	.	.	.	.	-	.	.	.	.	.	.	.
D. simulans	**A**	**T**	**A**	**.**	**A**	**.**	**T**	**C**	**.**	**G**	**.**	**A**	**-**	**.**	**T**	**A**	**-**	**.**	**-**	**.**	**.**	**-**	**.**	**G**

	3756	3765	3776	3937	4015	4049	4086	4124	4154	4196	4198	4231	4370 (E2)	4409 (E2)	4414 (E2)	4451 (E2)	4454 (E2)	4507	4534	4686 (E1)	4909	5217:d17	5618	5671:i	5774
**CNIII 1**	A	G	G	C	C	T	T	T	G	T	A	T	A	C	G	C	A	T	A	C	G	T	C	T	T
**CNIII 5**	.	.	.	.	.	.	.	.	.	.	.	.	.	T	A	.	G	C	G	T	.	.	.	.	.
**CNIII 6**	.	.	.	.	.	.	.	.	.	.	.	.	.	T	A	.	G	C	G	T	.	.	.	.	.
**CNIII 7**	.	.	.	.	.	.	.	.	.	.	.	.	.	T	A	.	G	C	G	T	.	d	.	A	.
**CNIII 15**	.	.	.	.	.	G	.	.	.	.	.	.	.	T	A	.	G	C	G	T	.	.	.	.	.
**CNIII 16**	T	A	A	.	.	.	.	A	.	.	.	.	.	T	A	.	G	C	G	T	.	.	.	.	.
**CNIII 18**	.	.	.	.	T	.	.	.	.	.	.	.	.	T	A	.	G	.	G	T	.	.	.	.	A
**CNIII 22**	.	.	.	.	.	.	.	.	.	.	.	.	.	T	A	.	G	C	G	T	.	.	A	.	.
**CNIII 35**	.	.	.	A	.	.	.	.	.	A	G	G	.	T	A	.	G	C	G	T	A	.	.	.	.
**CNIII 36**	.	.	.	A	T	.	C	.	A	A	.	.	T	.	A	T	G	C	G	T	.	.	.	.	.
D. simulans	**.**	**A**	**.**	**A**	**.**	**.**	**.**	**.**	**A**	**A**	**.**	**G**	**.**	**.**	**.**	**.**	**G**	**C**	**G**	**.**	**.**	**.**	**.**	**-**	**A**

	5777	6051	6086	6110	6200:d7	6235	6342:i2	6345	6488 (E2)	6530 (E2)	6551 (E2)	6578 (E2)	6620 (E2)	6807 (E1)	6840 (E1)	7027	7211	7701	7752	7791
**CNIII 1**	A	G	T	T	d	G	T	A	T	C	T	A	C	T	C	G	G	T	G	C
**CNIII 5**	.	.	.	.	G	.	-	G	.	.	.	.	.	.	.	.	.	C	A	.
**CNIII 6**	.	.	.	.	G	.	-	G	.	.	.	.	.	.	.	.	.	.	.	.
**CNIII 7**	.	.	.	.	G	.	-	G	.	.	.	.	.	C	.	.	.	.	.	.
**CNIII 15**	.	.	.	.	d	.	.	.	.	.	.	.	.	C	.	.	.	.	.	.
**CNIII 16**	.	.	.	.	G	.	-	G	.	.	.	.	.	.	.	T	.	.	.	.
**CNIII 18**	T	A	G	C	G	T	.	.	G	T	C	G	T	.	T	.	A	.	.	.
**CNIII 22**	.	.	.	.	G	.	.	.	.	.	.	.	.	.	.	.	.	.	.	.
**CNIII 35**	.	.	.	.	G	.	-	G	.	.	.	.	.	.	.	.	.	.	.	.
**CNIII 36**	.	.	.	.	G	.	-	G	.	.	.	.	.	.	.	.	.	C	A	G
D. simulans	**T**	**.**	**G**	**C**	**G**	**.**	**-**	**.**	**G**	**.**	**C**	**G**	**.**	**C**	**.**	**.**	**.**	**C**	**A**	**G**
