# Supplementary material for: Patterns of Nucleotide Diversity at the Regions Encompassing the Drosophila Insulin-Like Peptide (dilp) Genes: Demography vs. Positive Selection in Drosophila melanogaster
Source: PLoS One. 2013 Jan 7;8(1):e53593. doi: 10.1371/journal.pone.0053593 (PMC3538593; doi:10.1371/journal.pone.0053593)
Supplement: Figure S2 — (A) Genomic organization of the dilp5 gene region of D. melanogaster. Genomic DNA is represented by a line. The black arrow head points to the centromere. In gene, arrow indicates the direction of transcription. Colored boxes indicate exons of dilp5 gene. Intron are represented by a V symbol. (B) Nucleotide polymorphism at the dilp 5 gene region of D. melanogaster. The last row shows nucleotide information present in D. simulans for each polymorphic site detected in D. melanogaster. Dots indicate nucleotide variants identical to the first sequence and dashes indicate gaps. d, deletion; i, insertion; E, exon. (PDF) [file pone.0053593.s002.pdf]

A

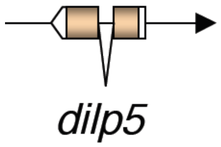

B

5'

	5	84	132	168	297	360	402	424	432	438	480	486	552	588	600	630	633	634	658	666	683	706	708	732	780	898	905	918	982	985	986	1066	1075	1084	1102	1128	1134:d2	1136	1141:d11	1155	1158	1161	1162	1164	1176
**CNIII 1**	G	T	A	T	C	T	A	G	T	C	T	G	A	G	T	T	C	d	A	-	T	G	-	T	A	C	T	A	A	A	A	C	T	A	d	A	d	-	d	d	A	C	G	T	C
**CNIII 5**	·	C	T	·	·	·	·	A	·	·	A	A	G	A	C	·	T	G	·	-	C	d	A	C	G	T	·	T	·	·	-	T	C	·	d	·	A	G	C	d	T	T	A	G	T
**CNIII 7**	A	·	·	C	T	G	G	·	C	T	·	A	G	A	C	·	-	G	·	-	C	d	A	C	G	T	G	·	·	·	-	·	C	·	d	·	A	G	d	T	·	T	A	·	·
**CNIII 9**	·	·	·	·	·	·	·	·	·	·	·	·	·	·	·	·	·	d	C	-	·	·	-	·	·	T	G	·	d	·	T	·	C	G	A	C	A	G	d	T	·	T	A	·	·
**CNIII 15**	·	·	·	C	·	·	·	A	·	·	A	A	G	A	C	d	T	G	·	-	C	d	A	C	G	T	·	T	·	·	-	·	C	·	d	·	A	G	C	d	T	T	A	G	T
**CNIII 18**	·	·	·	·	·	·	·	·	·	·	·	·	·	·	·	·	·	d	·	-	·	·	A	·	·	T	·	·	·	·	·	·	·	·	d	·	d	-	d	d	·	·	·	·	·
**CNIII 19**	·	·	·	·	·	·	·	·	·	·	·	·	·	·	·	·	·	d	·	-	·	·	-	·	·	T	G	·	d	·	T	·	C	G	A	C	A	G	d	T	·	T	A	·	·
**CNIII 22**	·	·	·	·	·	·	·	·	·	·	·	·	·	·	·	·	·	d	·	-	·	·	-	·	·	T	·	·	·	·	·	·	·	·	d	·	d	-	d	d	·	·	·	·	·
**CNIII 23**	·	A	·	C	T	G	G	·	C	T	·	A	G	A	C	d	T	G	·	-	C	d	A	C	·	T	·	T	·	·	-	·	C	·	d	·	A	G	C	d	T	T	A	G	T
**CNIII 34**	·	A	G	C	T	G	·	·	·	·	·	·	·	·	·	·	·	d	·	-	·	·	-	·	·	T	G	·	d	·	T	·	C	G	A	C	A	G	d	T	·	T	A	·	·
**CNIII 36**	A	·	·	C	T	G	G	·	C	T	·	A	·	·	·	d	T	C	·	-	·	d	G	·	·	T	G	·	·	-	T	·	C	·	d	·	A	G	C	d	T	T	A	G	T
**CNIII 41**	·	·	·	·	·	·	·	·	·	·	·	·	·	·	·	·	·	d	·	T	·	·	-	·	·	T	G	·	d	·	T	·	C	G	A	C	A	G	d	T	·	T	A	·	·
D. simulans	**·**	**C**	**G**	**C**	**·**	**G**	**G**	**·**	**A**	**·**	**·**	**·**	**·**	**·**	**C**	**·**	**-**	**G**	**·**	**-**	**C**	**·**	**-**	**C**	**G**	**T**	**G**	**·**	**·**	**-**	**-**	**·**	**C**	**G**	**A**	**·**	**A**	**-**	**C**	**T**	**·**	**T**	**A**	**·**	**·**

3'

	1188	1191	1209	1210	1220	1236	1237	1239	1266	1272	1281	1303	1317	1321	1349	1355:d2	1357:d6	1386	1451	1454	1458:d25	1463	1472	1476	1649	1764	E1 1851	2498	2507	2545	2579	2684	2754	2818	2873	2874	2881	2905	2929	2959	2982	2986	3059	3081:i4	3156
**CNIII 1**	C	G	A	T	G	T	C	G	C	T	C	A	A	C	A	T	T	A	A	C	T	G	A	G	G	G	C	T	C	-	A	A	A	C	C	C	T	A	T	C	T	C	A	-	A
**CNIII 5**	T	A	G	C	C	G	T	A	T	C	G	G	T	A	·	·	·	·	·	·	·	·	·	·	·	·	·	·	·	T	G	·	G	·	·	·	·	G	·	T	·	·	T	-	C
**CNIII 7**	·	·	·	·	·	·	A	·	·	·	G	·	·	·	G	d	d	·	C	d	d	—	—	—	A	A	A	·	·	T	G	·	G	T	T	T	A	G	·	·	·	T	T	-	C
**CNIII 9**	·	·	·	·	·	·	·	·	·	·	·	·	·	·	·	·	·	·	·	·	·	·	·	·	·	·	·	·	·	T	G	·	G	T	T	T	A	G	·	·	·	T	T	-	C
**CNIII 15**	T	A	G	C	C	G	T	A	T	C	G	G	T	A	·	·	·	·	·	·	·	·	·	·	·	·	·	·	·	T	G	·	G	·	·	·	·	G	·	T	·	·	T	-	C
**CNIII 18**	·	·	·	·	·	·	·	·	·	·	·	·	·	·	·	·	·	·	·	·	·	·	·	·	·	·	·	·	·	T	G	·	·	·	·	·	·	·	·	·	·	·	·	-	·
**CNIII 19**	·	·	·	·	·	·	·	·	·	·	·	·	·	·	·	·	·	·	·	·	·	·	·	·	·	·	·	·	·	-	G	·	·	·	·	·	·	·	·	·	·	·	·	-	C
**CNIII 22**	·	·	·	·	·	·	·	·	·	·	·	·	·	·	·	·	·	·	·	·	·	·	·	·	·	·	·	·	·	T	G	·	·	·	·	·	·	·	·	·	·	·	·	-	·
**CNIII 23**	T	A	G	C	C	G	T	A	T	C	G	G	T	A	·	d	·	G	·	·	·	A	T	A	A	·	·	d	G	-	G	·	G	·	·	·	·	G	·	T	C	·	T	-	C
**CNIII 34**	·	·	·	·	·	·	·	·	·	·	·	·	·	·	·	·	·	·	·	·	·	·	·	·	·	·	·	·	·	-	G	·	·	·	·	·	·	·	·	·	·	·	·	-	·
**CNIII 36**	T	A	G	C	C	G	T	A	T	C	G	G	T	A	·	d	·	G	·	·	·	A	T	A	·	·	·	·	·	-	G	G	G	·	·	·	·	G	A	·	·	·	T	A	C
**CNIII 41**	·	·	·	·	·	·	·	·	·	·	·	·	·	·	·	·	·	·	·	·	·	·	·	·	·	·	·	·	·	-	G	·	·	·	·	·	·	·	·	·	·	·	·	-	C
D. simulans	**·**	**·**	**C**	**·**	**C**	**G**	**T**	**·**	**·**	**-**	**G**	**G**	**·**	**T**	**·**	**·**	**·**	**C**	**·**	**·**	**·**	**·**	**T**	**·**	**·**	**·**	**·**	**·**	**·**	**-**	**G**	**G**	**-**	**·**	**·**	**·**	**·**	**G**	**·**	**·**	**·**	**·**	**G**	**-**	**C**
