# Supplementary material for: Patterns of Nucleotide Diversity at the Regions Encompassing the Drosophila Insulin-Like Peptide (dilp) Genes: Demography vs. Positive Selection in Drosophila melanogaster
Source: PLoS One. 2013 Jan 7;8(1):e53593. doi: 10.1371/journal.pone.0053593 (PMC3538593; doi:10.1371/journal.pone.0053593)
Supplement: Figure S3 — (A) Genomic organization of the dilp6 and dilp7 gene regions of D. melanogaster. Genomic DNA is represented by a line. The black arrow head points to the centromere. In genes, arrows indicate the direction of transcription. Colored boxes indicate exons of dilp genes Introns are represented by a V symbol. (B) Nucleotide polymorphism at the dilp6 and dilp7 gene regions of D. melanogaster. The last row shows nucleotide information present in D. simulans for each polymorphic site detected in D. melanogaster. *, nonsynonymous polymorphism. Dots indicate nucleotide variants identical to the first sequence and dashes indicate gaps. i, insertion; E, exon. (PDF) [file pone.0053593.s003.pdf]

A

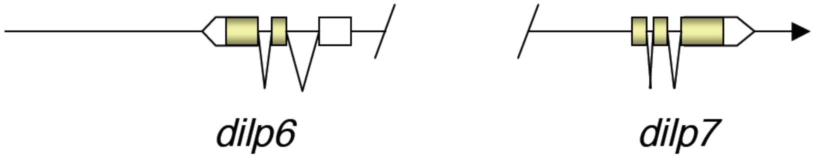

B

	5'	E1																										//		E2				3'			
	12	232	254	353	359	426	485	520	572	606	681	781	782	827	852:i4	915	918	920	922	965	986	988	1017	1078	1199	1228	1229		1638	2000 *	2100	2124:i8	2132:i8	2177	2494:i2	2525	2726
**CNX 3**	C	A	A	G	A	T	A	G	-	-	C	T	C	T	C	T	T	T	C	T	T	G	A	A	C	C	G		C	C	C	G	-	T	-	T	T
**CNX 4**	.	.	.	.	.	.	.	.	-	-	.	.	.	A	.	.	.	.	.	.	.	A	.	.	.	.	.		.	.	G	-	-	C	-	.	.
**CNX 5**	.	.	G	A	.	A	G	.	-	A	A	.	.	.	.	.	.	.	.	.	.	.	.	.	.	.	.		.	.	.	.	-	C	-	.	.
**CNX 7**	.	.	G	A	.	A	G	.	-	A	A	G	.	.	.	.	.	.	.	.	.	.	G	.	.	.	.		.	.	.	.	-	.	-	.	A
**CNX 10**	T	.	G	A	.	A	G	.	-	A	A	.	.	.	.	.	.	.	.	.	.	.	.	.	.	G	C		.	.	.	.	-	.	-	.	.
**CNX 11**	.	.	.	.	.	.	.	.	-	A	.	.	.	A	-	.	.	.	.	.	.	.	.	-	.	.	.		.	.	.	.	-	.	-	.	.
**CNX 13**	.	.	G	A	.	A	G	.	-	A	A	.	.	.	.	.	.	.	.	G	G	.	.	.	.	.	.		.	G	.	-	-	.	T	C	A
**CNX 15**	.	.	G	A	.	A	G	.	-	A	A	.	.	.	.	.	.	.	.	.	.	.	.	.	.	.	.		d	.	G	-	-	C	-	.	A
**CNX 16**	.	.	G	A	.	A	G	.	-	A	A	G	.	.	.	.	.	.	.	.	.	.	.	.	.	.	.		.	.	.	.	-	.	-	.	A
**CNX 17**	.	G	.	.	T	.	.	.	T	-	.	.	.	.	.	.	.	.	.	.	.	.	.	.	.	.	.		.	.	.	.	A	.	T	.	A
**CNX 19**	.	.	G	A	.	A	G	T	-	A	A	.	T	.	.	G	G	G	.	.	.	A	.	.	.	.	.		d	.	G	-	-	C	-	.	.
**CNX 21**	.	.	G	A	.	A	G	.	-	T	.	.	.	.	.	G	G	G	G	.	.	.	.	.	A	.	.		.	.	.	.	-	.	-	.	.
D. simulans	**-**	**.**	**G**	**.**	**.**	**A**	**.**	**A**	**-**	**-**	**.**	**.**	**.**	**.**	**-**	**.**	**.**	**.**	**.**	**.**	**.**	**.**	**.**	**-**	**.**	**.**	**.**		**.**	**.**	**-**	**-**	**-**	**C**	**-**	**C**	**-**

			E1			E2							E3			
	19	33	121	209	265	330	339	453:i3	497	507	508:i8	537	713	725	727 *	728
**CNX 3**	C	A	A	C	A	T	C	-	A	A	-	T	T	C	A	T
**CNX 4**	·	·	G	T	T	C	·	G	G	C	A	A	C	G	·	·
**CNX 5**	·	G	G	T	T	C	·	G	G	C	A	A	C	·	·	·
**CNX 7**	·	G	G	T	T	C	·	G	G	C	A	A	C	·	·	·
**CNX 10**	G	·	G	T	T	C	·	G	G	C	A	A	C	·	·	·
**CNX 11**	·	·	G	T	T	C	·	G	G	C	A	A	C	·	·	·
**CNX 13**	·	G	G	T	T	C	·	G	G	C	A	A	·	·	·	·
**CNX 15**	·	G	G	T	T	C	·	G	G	C	A	A	C	·	·	·
**CNX 16**	·	·	G	T	T	C	·	G	G	C	A	A	C	·	T	C
**CNX 17**	·	G	G	·	·	C	·	-	·	·	-	·	·	·	·	·
**CNX 19**	·	G	·	·	T	C	A	G	G	C	A	A	C	·	·	·
**CNX 21**	·	G	G	T	T	C	·	G	G	C	A	A	C	·	·	·
D. simulans	**·**	**G**	**G**	**·**	**T**	**C**	**·**	**-**	**-**	**-**	**-**	**G**	**C**	**·**	**·**	**·**
