# Supplementary material for: Patterns of Nucleotide Diversity at the Regions Encompassing the Drosophila Insulin-Like Peptide (dilp) Genes: Demography vs. Positive Selection in Drosophila melanogaster
Source: PLoS One. 2013 Jan 7;8(1):e53593. doi: 10.1371/journal.pone.0053593 (PMC3538593; doi:10.1371/journal.pone.0053593)
Supplement: Figure S4 — (A) Schematic representation of the predicted structure of the DILP1-5 proteins of D. melanogaster. SP, signal peptide; B, B chain; C, C peptide; A, A chain. The active peptide chains are denoted by colors. (B) Amino acid polymorphism in D. melanogaster and amino acid replacements between D. melanogaster and D. simulans at the DILP1-5 proteins. The last row shows the amino acid present in D. simulans for each polymorphic site detected in D. melanogaster and also for the sites with fixed differences between species. Dots indicate amino acid variants identical to the first sequence and dashes indicate deletions. (PDF) [file pone.0053593.s004.pdf]

A

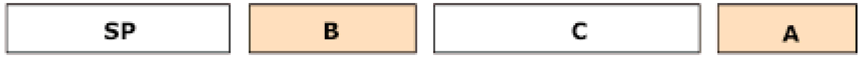

B

	DILP1														
	SP	B		C											
	10	31	39	77	78	83	86	87	90	91	94	103	123	126	127
**CNIII 1**	V	V	Q	-	G	D	T	E	V	E	S	A	M	H	-
**CNIII 5**	.	.	.	-	.	.	.	.	.	Q	.	.	.	.	-
**CNIII 6**	.	.	.	-	.	.	.	.	.	Q	.	.	.	.	-
**CNIII 7**	.	.	.	-	.	.	.	.	.	Q	.	.	.	.	-
**CNIII 15**	.	.	.	-	.	.	.	.	.	Q	.	.	.	.	-
**CNIII 16**	.	.	.	-	.	.	.	.	.	Q	.	.	.	.	-
**CNIII 18**	.	A	.	-	.	G	A	.	.	.	T	.	.	.	-
**CNIII 22**	.	.	.	-	.	.	.	.	.	Q	.	.	.	.	-
**CNIII 35**	.	.	.	-	.	.	.	.	.	Q	.	.	.	.	-
**CNIII 36**	.	.	.	-	.	.	.	.	.	Q	.	.	.	.	-
D. simulans	**A**	**A**	**H**	**L**	**A**	**.**	**A**	**K**	**E**	**.**	**.**	**T**	**T**	**R**	**Y**

	DILP2			DILP3		DILP4					
	SP	C		SP	C	SP		C			
	5	63	99	9	65	11	14	55	76	88	102
**CNIII 1**	L	A	N	D	Q	L	L	S	I	K	V
**CNIII 5**	.	.	.	.	.	.	.	.	.	.	.
**CNIII 6**	.	.	.	.	.	.	.	.	.	.	.
**CNIII 7**	.	.	.	.	.	.	.	.	.	.	.
**CNIII 15**	.	.	.	.	.	.	.	.	.	.	.
**CNIII 16**	.	.	.	.	.	.	.	.	.	.	.
**CNIII 18**	.	.	.	.	.	.	.	.	.	.	.
**CNIII 22**	.	.	.	.	.	.	.	.	.	.	.
**CNIII 35**	.	.	.	.	.	.	.	.	.	.	.
**CNIII 36**	.	.	.	.	.	.	.	.	.	.	.
D. simulans	**V**	**P**	**S**	**G**	**L**	**R**	**M**	**T**	**T**	**Q**	**G**

	DILP5	
	C	A
	53	98
**CNIII 1**	T	F
**CNIII 5**	.	.
**CNIII 7**	.	.
**CNIII 9**	.	.
**CNIII 15**	.	.
**CNIII 18**	.	.
**CNIII 19**	.	.
**CNIII 22**	.	.
**CNIII 23**	.	.
**CNIII 34**	.	.
**CNIII 36**	.	.
**CNIII 41**	.	.
D. simulans	**S**	**L**
