# Supplementary material for: Patterns of Nucleotide Diversity at the Regions Encompassing the Drosophila Insulin-Like Peptide (dilp) Genes: Demography vs. Positive Selection in Drosophila melanogaster
Source: PLoS One. 2013 Jan 7;8(1):e53593. doi: 10.1371/journal.pone.0053593 (PMC3538593; doi:10.1371/journal.pone.0053593)
Supplement: Figure S5 — (A) Schematic representation of the predicted structure of the DILP6 and DILP7 proteins of D. melanogaster. SP, signal peptide; B, B chain; C, C peptide; A, A chain. The active peptide chains are denoted by colors. (B) Amino acid polymorphism in D. melanogaster and amino acid replacements between D. melanogaster and D. simulans at the DILP6 and DILP7 proteins. The last row shows the amino acid present in D. simulans for each polymorphic site detected in D. melanogaster and also for the sites with fixed differences between species. Dots indicate amino acid variants identical to the first sequence and dashes indicate deletions. (PDF) [file pone.0053593.s005.pdf]

A

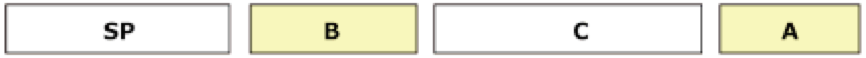

B

	DILP6						DILP7		
	SP				A		SP	C	A
	21	23	25	28	85	87	15	97	156
**CNX 3**	A	M	S	M	N	T	V	T	N
**CNX 4**	.	.	.	.	.	.	.	.	.
**CNX 5**	.	.	.	.	.	.	.	.	.
**CNX 7**	.	.	.	.	.	.	.	.	.
**CNX 10**	.	.	.	.	.	.	.	.	.
**CNX 11**	.	.	.	.	.	.	.	.	.
**CNX 13**	.	.	.	.	.	S	.	.	.
**CNX 15**	.	.	.	.	.	.	.	.	.
**CNX 16**	.	.	.	.	.	.	.	.	I
**CNX 17**	.	.	.	.	.	.	.	.	.
**CNX 19**	.	.	.	.	.	.	.	.	.
**CNX 21**	.	.	.	.	.	.	.	.	.
D. simulans	**P**	**L**	**G**	**V**	**S**	**.**	**L**	**S**	**.**
