# Supplementary material for: Patterns of Nucleotide Diversity at the Regions Encompassing the Drosophila Insulin-Like Peptide (dilp) Genes: Demography vs. Positive Selection in Drosophila melanogaster
Source: PLoS One. 2013 Jan 7;8(1):e53593. doi: 10.1371/journal.pone.0053593 (PMC3538593; doi:10.1371/journal.pone.0053593)
Supplement: Table S1 — Nucleotide polymorphism and divergence at the autosomal dilp1-4 and dilp5 gene regions. (PDF) [file pone.0053593.s006.pdf]

Nucleotide polymorphism and divergence at the autosomal *dilp1-4* and *dilp5* gene regions

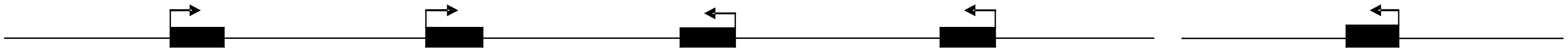

	**5'**	***dilp1***	intergenic	***dilp2***	intergenic	***dilp3***	intergenic	***dilp4***	**3'**	**3'**	***dilp5***	**5'**
**No. sites**												
Intronic	n. a.	n. a.	n. a.	73	n. a.	72	n. a.	61	n. a.	n. a.	71	n. a.
Synonymous	n. a.	110.3	n. a.	98.5	n. a.	85.8	n. a.	103.1	n. a.	n. a.	80.2	n. a.
Silent	907	110.3	868	171.5	1326	157.8	1577	164.1	899	1092	151.2	1717
Non-synonymous	n. a.	351.7	n. a.	312.5	n. a.	274.2	n. a.	298.9	n. a.	n. a.	240.8	n. a.
Total	907	462	868	484	1326	432	1577	463	899	1092	392	1717
S												
Intronic	n. a.	n. a.	n. a.	2 (1)	n. a.	2(1)	n. a.	0	n. a.	n. a.	0	n. a.
Synonymous	n. a.	1 (1)	n. a.	6 (6)	n. a.	6 (5)	n. a.	7 (6)	n. a.	n. a.	1 (1)	n. a.
Silent	1 (1)	1 (1)	10 (8)	8 (7)	41 (36)	8 (6)	10 (9)	7 (6)	5 (3)	15 (5)	1 (1)	50 (6)
Non-synonymous	n. a.	5 (4)	n. a.	0	n. a.	0	n. a.	0	n. a.	n. a.	0	n. a.
Total	1 (1)	6 (5)	10 (8)	8 (7)	41 (36)	8 (6)	10 (9)	7 (6)	5 (3)	15 (5)	1 (1)	50 (6)
π												
Intronic	n. a.	n. a.	n. a.	0.008	n. a.	0.008	n. a.	0	n. a.	n. a.	0	n. a.
Synonymous	n. a.	0.002	n. a.	0.012	n. a.	0.016	n. a.	0.015	n. a.	n. a.	0.002	n. a.
Silent	0.0002	0.002	0.003	0.010	0.007	0.012	0.001	0.009	0.001	0.004	0.001	0.012
Non-synonymous	n. a.	0.003	n. a.	0	n. a.	0	n. a.	0	n. a.	n. a.	0	n. a.
Total	0.0002	0.003	0.003	0.004	0.007	0.004	0.001	0.003	0.001	0.004	0.0004	0.012
h	2	3	6	5	9	4	6	3	5	7	2	10
Hd	0.20	0.38	0.78	0.67	0.98	0.53	0.84	0.51	0.67	0.90	0.17	0.97
K												
Intronic	n. a.	n. a.	n. a.	0.082	n. a.	0.054	n. a.	0.051	n. a.	n. a.	0.030	n. a.
Synonymous	n. a.	0.116	n. a.	0.143	n. a.	0.197	n. a.	0.159	n. a.	n. a.	0.094	n. a.
Silent	0.049	0.116	0.026	0.116	0.081	0.133	0.052	0.117	0.043	0.041	0.064	0.054
Non-synonymous	n. a.	0.031	n. a.	0.010	n. a.	0.007	n. a.	0.020	n. a.	n. a.	0.008	n. a.
Total	0.049	0.051	0.026	0.046	0.081	0.049	0.052	0.053	0.043	0.041	0.029	0.054

S, number of segregating sites (number of singletons in parentheses); *π*, nucleotide diversity; *h*, number of haplotypes; *Hd*, haplotype diversity; *K*, nucleotide divergence; n. a., not applicable.
