# Supplementary material for: Patterns of Nucleotide Diversity at the Regions Encompassing the Drosophila Insulin-Like Peptide (dilp) Genes: Demography vs. Positive Selection in Drosophila melanogaster
Source: PLoS One. 2013 Jan 7;8(1):e53593. doi: 10.1371/journal.pone.0053593 (PMC3538593; doi:10.1371/journal.pone.0053593)
Supplement: Table S2 — Nucleotide polymorphism and divergence at the X-linked dilp6 and dilp7 gene regions. (PDF) [file pone.0053593.s007.pdf]

Nucleotide polymorphism and divergence at the X-linked *dilp6* and *dilp7* gene regions

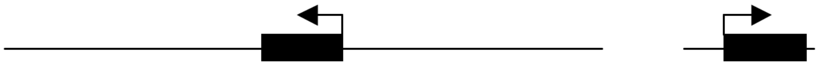

	3'	*dilp6*	5'	*dilp7*
**No. sites**				
Intronic	n. a.	870	n. a.	170
Synonymous	n. a.	75.8	n. a.	112.1
Silent	766	1312.8	29	334.1
Non-synonymous	n. a.	245.2	n. a.	364.9
Total	766	1558	29	669
S				
Intronic	n. a.	18 (10)	n. a.	5 (0)
Synonymous	n. a.	0	n. a.	6 (4)
Silent	4 (1)	22 (11)	1 (1)	13 (5)
Non-synonymous	n. a.	1 (1)	n. a.	1 (1)
Total	4 (1)	23 (12)	1 (1)	14 (6)
π				
Intronic	n. a.	0.006	n. a.	0.01
Synonymous	n. a.	0	n. a.	0.012
Silent	0.002	0.005	0.006	0.011
Non-synonymous	n. a.	0.0007	n. a.	0.0005
Total	0.002	0.004	0.006	0.005
h	6	11	2	9
Hd	0.85	0.98	0.17	0.91
K				
Intronic	n. a.	0.062	n. a.	0.166
Synonymous	n. a.	0.177	n. a.	0.118
Silent	0.043	0.071	n. a.	0.164
Non-synonymous	n. a.	0.021	n. a.	0.010
Total	0.043	0.062	n. a.	0.073

S, number of segregating sites (number of singletons in parentheses); π, nucleotide diversity; *h*, number of haplotypes; *Hd*, haplotype diversity; *K*, nucleotide divergence; n. a., not applicable.
